# Comparison of the clinical characteristics and prognosis of primary versus secondary acute gastrointestinal injury in critically ill patients

**DOI:** 10.1186/s40560-017-0221-4

**Published:** 2017-04-20

**Authors:** Dong Zhang, Rao Fu, Yanhua Li, Hongyan Li, Yuting Li, Hongxiang Li

**Affiliations:** grid.430605.4 ICU, The First Hospital of Jilin University, No. 71 Xinmin Street, Changchun, China

**Keywords:** Acute gastrointestinal injury, Primary, Secondary, Classification system, Mortality

## Abstract

**Background:**

This prospective study compared clinical characteristics and prognosis between primary (P) and secondary (S) acute gastrointestinal injury (AGI) (P-AGI)/(S-AGI) in critically ill patients.

**Methods:**

This was a prospective, single-center observational study. Patients were included if they had been hospitalized for at least 72 h before the AGI diagnosis. Patients were classified according to severity of gastrointestinal dysfunction, while P-AGI or S-AGI were defined according to whether the gastrointestinal system was directly or indirectly involved. Clinical characteristics, Acute Physiology and Chronic Health Evaluation (APACHE) II score, and Sepsis-related Organ Failure Assessment (SOFA) scores after inclusion and 28-day mortality were recorded.

**Results:**

Altogether, 282 patients were included: P and S groups enrolled 100 and 182 patients, respectively. The S group patients were older and showed increased morbidities and higher APACHE II and SOFA scores. Compared to the S group, the P group had a higher prevalence in abdominal distention and enteroparalysis and fewer patients at AGI grade I, while more patients at grade III or IV. The S group patients had the higher 28-day mortality. Multiple logistic regression analysis showed AGI grades, APACHE II score, and S-AGI independently predicted the odds of 28-day mortality.

**Conclusions:**

Comparing to the P-AGI patients, the S group patients were older, with higher APACHE II and SOFA scores. AGI grade, APACHE II score, and S-AGI independently predicted the odds of 28-day mortality in AGI patients.

## Background

The gastrointestinal (GI) tract is vulnerable in critically ill patients and GI dysfunction is common with morbidity as high as 50% [1, 2]. The intestine plays an important role in the development of multiple organ dysfunction syndrome (MODS) [3]. Early diagnosis and therapy to GI dysfunction could improve prognosis of critically ill patients [4].

In 2012, The Working Group on Abdominal Problems (WGAP) of the European Society of Intensive Care Medicine (ESICM) proposed a definition for acute gastrointestinal injury (AGI) and recommended a four-grade classification system for AGI severity [5]. According to the guidelines, AGI can be defined as primary AGI (P-AGI) or secondary AGI (S-AGI) [5]. However, whether these definitions and associated four-grade severity classification are helpful in diagnosis remains unclear. The aim of the present study was to compare the clinical characteristics and prognosis according to new definitions and severity classifications.

## Methods

### Study design

This prospective, observational study was conducted in a general intensive care unit (ICU) at the First Hospital of Jilin University (Changchun, China) from 1 January 2014 to 30 June 2015.

### Patient selection and grouping

Patients were included if they had been hospitalized for at least 72 h before being diagnosed with AGI according to the ESICM definition [5]. Patients were excluded from the present study if they were under 18 years old; diagnosed with a malignancy; suffered from Crohn’s disease, ulcerative colitis, or short bowel syndrome; or were hospitalized for less than 72 h before AGI diagnosis was established. Severity grades of AGI were distinguished according to ESICM criteria: AGI grade I (risk of developing GI dysfunction or failure): the function of the GI tract was partially impaired, expressed as GI symptoms related to a known cause and perceived as transient. AGI grade II (GI dysfunction): the GI tract was not able to perform digestion and absorption adequately to satisfy the nutrient and fluid requirements of the body. There were no changes in the general condition of the patient related to GI problems. AGI grade III (GI failure): loss of GI function, where restoration of GI function was not achieved despite interventions and the general condition was not improving. AGI grade IV (GI failure with severe impact on distant organ function): AGI had progressed to become directly and immediately life-threatening, with worsening of MODS and shock. P-AGI was associated with primary disease or direct injury to organs of the GI system, such as peritonitis, pancreatitis, or abdominal surgery. S-AGI developed as the consequence of a host response in critical illness without primary pathology in the GI system, such as GI malfunction in pneumonia or non-abdominal surgery [5]. In ESICM criteria, feeding intolerance should be considered present if at least 20 kcal/kg BW/day via enteral route could not be reached within 72 h of feeding attempt or if enteral feeding had to be stopped for whatever clinical reason. Gastric residual volume could be considered high if a single volume exceeds 200 ml.

### Data collection and clinical evaluation

The following data were acquired: demographic details; AGI grade; intra-abdominal pressure (IAP) (the highest value obtained on bladder manometry in the first 3 days, with each measurement being performed at a fixed time point; measurements were performed at least four times per day, with mean values calculated [6]); abdominal perfusion pressure (APP; difference between mean blood pressure and IAP); Acute Physiology and Chronic Health Evaluation (APACHE) II score (in the first 24 h after ICU admission); Sepsis-related Organ Failure Assessment (SOFA) score (in the first 24 h after ICU admission); and 28-day mortality.

### Statistical analyses

Categorical variables are presented as percentages, whereas continuous variables are presented as median and interquartile range (IQR). Categorical variables were compared using the chi-squared test or Fisher test and continuous variables using the Mann–Whitney *U* test. Variables (age, gender, AGI grade, APACHE II score, and S-AGI) were included in the multiple logistic regression analysis of the 28-day mortality. A *P* value of <0.05 was considered statistically significant. All tests were two-sided. Data were analyzed using commercially available software (PASW Statistics, version 17.0; SPSS, Chicago, IL, USA).

## Results

### Patient enrollment

There were 322 patients initially enrolled: 20 patients had no IAP measurements, 9 patients lost follow-up, and 11 patients were with unclear AGI classification. Thus, 282 patients were finally included: 190 males (67.4%) and 92 females (32, 6%), with a median age of 66 years (IQR 48–78 years), a median APACHE II score of 17 (IQR 12–23), and a median SOFA score of 6 (IQR 4–9) (Table 1).

**Table 1:** Characteristics of primary and secondary AGI in critically ill patients

Characteristics	P-AGI (*n* = 100)	S-AGI (*n* = 182)	All (*n* = 282)	*P* value
Age, years	61 (42–72)	70 (55–81)	66 (48–79)	<0.001
Male, no. (%)	67 (67%)	123 (67.6%)	190 (67.4%)	0.921
BMI (kg/m^2^)	23.3 (20.9–25.8)	23.4 (21.2–24.7)	23.3 (21.2–25.0)	0.776
APACHE II score	15 (9–20)	18 (13–25)	17.0 (12–23)	<0.001
SOFA score	5 (3–8)	6 (4–10)	6 (4–9)	0.016
Etiology of AGI				
AP	36			
Peritonitis	22			
Abdominal surgery or trauma	42			
Pneumonia		49		
Non-abdominal surgery or trauma		32		
Post-resuscitation		20		
Shock		51		
Sepsis		30		
Comorbidities				
Hypertension, no. (%)	40 (40%)	95 (52.2%)	145 (51.4%)	0.050
Diabetes, no. (%)	20 (20%)	52 (28.6%)	72 (25.5%)	0.114
CHF, no. (%)	4 (4%)	32 (17.6%)	36 (12.8%)	0.001
CVD, no. (%)	3 (3%)	35 (19.2%)	38 (13.5%)	<0.001
COPD, no. (%)	3 (3%)	29 (15.9%)	32 (11.3%)	0.001
CKD, no. (%)	4 (4%)	19 (10.4%)	23 (8.2%)	0.059

*AP* acute pancreatitis, *BMI* body mass index, *APACHE II* Acute Physiology and Chronic Health Evaluation II, *CHF* chronic heart failure, *CKD* chronic kidney disease, *COPD* chronic obstructive pulmonary disease, *CVD* cerebrovascular disease, *SOFA* Sepsis-related Organ Failure Assessment

### Characteristics of primary and secondary AGI in critically ill patients

One hundred P group and 182 S group patients were enrolled in our study. The S group patients were older, with a higher APACHE II and SOFA score and with higher prevalence of comorbidities such as chronic heart failure (CHF), cerebrovascular disease (CVD), and chronic obstructive pulmonary disease (COPD) (Table 1).

### Comparison of clinical manifestations in primary and secondary AGI patients

Abdominal distention and enteroparalysis were more prevalent in P group patients than in S group patients. There was no difference in hypoactive bowel sounds, feeding intolerance, gastric retention, diarrhea, and GI bleeding between the two groups (Table 2).

**Table 2:** Comparison of primary and secondary AGI in clinical manifestation about GI tract

Parameters of digestive tract	P-AGI (*n* = 100)	S-AGI (*n* = 182)	*P* value
Hypoactive bowel sounds, no. (%)	86 (86.0%)	146 (80.2%)	0.224
Abdominal distention, no. (%)	84 (84.0%)	126 (69.2%)	0.007
Feeding intolerance^a^, no. (%)	56 (56.0%)	83 (45.6%)	0.095
Enteroparalysis, no. (%)	48 (48.0%)	50 (27.5%)	0.001
Gastric retention	23 (23.0%)	46 (25.3%)	0.671
Diarrhea, no. (%)	12 (12.0%)	31 (17.0%)	0.261
Positive in fecal occult test, no. (%)	18 (18.0%)	38 (20.9%)	0.562
GI bleeding, no. (%)	11 (11.0%)	16 (8.8%)	0.546
IAP >12 mmHg, no. (%)	22 (22.0%)	31 (17.0%)	0.340

*IAP*, intra-abdominal pressure, *GI* gastrointestinal, *P* primary, *S* secondary

^a^Feeding intolerance was considered to be present if the minimum of 20 kcal/kg BW/day via enteral route could not be reached within 72 h of feeding attempt or if enteral feeding had to be stopped for whatever clinical reason

### Comparison of primary and secondary AGI in severity and prognosis of AGI

Compared to the S group patients, the P group had less patients at AGI grade I but significantly more patients at grade III or IV (Table 3). In the different severity categories, only at grade II did the S group demonstrate a higher mortality. In general, the S group patients had a significantly higher 28-day mortality (Table 4). In the multiple logistic regression analysis, AGI grade, APACHE II score, and S-AGI independently predicted the odds of 28-day death (*P* < 0.05) (Table 5).

**Table 3:** Comparison of primary and secondary AGI in the severity of AGI

AGI grades	P-AGI (*n* = 109)	S-AGI (*n* = 182)	*P* value
I, *n* (%)	5 (5%)	53 (29.1%)	<0.001
II, *n* (%)	55 (55%)	109 (59.9%)	0.426
III, *n* (%)	26 (26%)	14 (7.7%)	<0.001
IV, *n* (%)	14 (14%)	6 (3.3%)	0.001

*AGI* acute gastrointestinal injury, *P* primary, S secondary

**Table 4:** Comparison of primary and secondary AGI in 28-day mortality of AGI

AGI grades	P-AGI	S-AGI	*P* value
I, *n* (%)	0	12/53 (22.6%)	0.573
II, *n* (%)	2/55 (3.6%)	27/109 (24.8%)	<0.001
III, *n* (%)	8/26 (30.8%)	8/14 (57.1%)	0.176
IV, *n* (%)	7/14 (50.0%)	4/6 (66.7%)	0.642
Total, *n* (%)	17 (17%)	51 (28%)	0.038

*AGI* acute gastrointestinal injury, *P* primary, *S* secondary

**Table 5:** Comparison 28-day mortality of AGI with primary and secondary AGI in total patients

	OR (95% CI)	*P* value
Age	1.008 (0.990–1.026)	0.370
Male	0.730 (0.381–1.400)	0.344
AGI grade	2.276 (1.503–3.446)	<0.001
APACHE II score	1.093 (1.047–1.141)	<0.001
S-AGI	2.656 (1.202–5.870)	0.016

*AGI* acute gastrointestinal injury, *APACHE II* Acute Physiology and Chronic Health Evaluation II, *S* secondary

## Discussion

The present study showed that the S group patients were older, with higher APACHE II and SOFA scores and with a higher prevalence of comorbidities, but less severe AGI compared to the P group patients. AGI grade, APACHE II score, and S-AGI independently predicted the odds of 28-day mortality.

In the P group patients, acute pancreatitis, peritonitis, and abdominal surgery/trauma were the principal causes of AGI. These diseases lead to GI injury directly and usually increase IAP [7–9], which may account for the P group patients demonstrating symptoms of abdominal distention and enteroparalysis more prevalently than the S group patients. Indeed, there were more grade III and IV AGI patients in the P group than in the S group. Previous studies showed that patients with abdominal injury had higher IAP than those without intra-peritoneal injury [10], which could explain why P-AGI was prone to intra-abdominal hypertension and more severe AGI in our study.

The S group patients showed a higher prevalence of comorbidities, such as CHF, CVD, and COPD. Indeed, such comorbidities may potentially provoke AGI development: CHF reduces bowel perfusion and impairs function of the intestinal barrier [11], CVD results in GI stress and complications following stroke [12], and COPD patients often suffer hypoxia [13] which impairs gut mucosa perfusion and GI function [14]. In addition, the S-AGI patients were older and had decreased GI reserve, which made them highly sensitive to minor insults, and decompensation could rapidly occur [15]. Therefore, these comorbidities might be helpful to explain the worse baseline GI function with S-AGI patients. On the base of it, severe injuries, such as trauma and shock, often developed hypotension, resulting in easy GI hypoperfusion and AGI, as the gut mucosa has a large surface area for absorption (approximately 100 m^2^) and is metabolically active and receives over half of the cardiac output [11]. Ischemia accounts for a major cause of acute GI mucosa lesions and GI injury [16], followed by systematic inflammatory response, which may damage endothelial glycocalyx, alter endothelial permeability, and impair GI microcirculation and perfusion [17, 18].

The proportions of different AGI grades differed between the P and S groups: there was a higher prevalence of grade III and IV patients in the P group than in the S group, which contributed to the higher severity in the S group. It is logical to postulate higher mortality in the P group. However, our results showed exactly the contrary. S-AGI with AGI II showed higher mortality; furthermore, there was a tendency of higher mortality in patients with S-AGI with AGI I, III, and IV, which was speculated partially because of older age, more comorbidities, and higher APACHE II/SOFA score with S-AGI patients. And multiple regression analysis showed not only high AGI grade or high APACHE II score but also S-AGI increased independently the odds of 28-day mortality. Only considering of different AGI grades was not enough to predict the prognosis, S-AGI could also independently predict mortality in AGI patients. OR: Not only was considering different AGI grades predict the prognosis, but S-AGI could independently predict mortality in AGI patients.

However, there are some limitations to our study. Firstly, complicated manifestations made AGI diagnosis and classifications difficult even following ESICM’s criteria, which potentially biased the outcome; secondly, the shortage of data regarding treatment conditions and methods of nutritional support made the risk factors insufficient as there was no adjustment for other potential confounding factors. Lastly, our single-center observational study with limited number of patients limited general extrapolation.

## Conclusions

Compared to P-AGI patients, S-AGI patients were generally older in age, with higher APACHE II and SOFA scores, and with more associated comorbidities. AGI grade, APACHE II score, and S-AGI were variables that independently predictable the odds of 28-day mortality in AGI patients.

## Abbreviations

AGI: Acute gastrointestinal injury
AP: Acute pancreatitis
APACHE II: Acute Physiology and Chronic Health Evaluation II
BMI: Body mass index
CHF: Chronic heart failure
CI: Confidence interval
CKD: Chronic kidney disease
COPD: Chronic obstructive pulmonary disease
CVD: Cerebrovascular disease
ESICM: European Society of Intensive Care Medicine
GI: Gastrointestinal
IQR: Interquartile range
P: Primary
S: Secondary
SOFA: Sepsis-related Organ Failure Assessment
WGAP: Working Group on Abdominal Problems
